# Tailoring motivational health messages for smoking cessation using an mHealth recommender system integrated with an electronic health record: a study protocol

**DOI:** 10.1186/s12889-018-5612-5

**Published:** 2018-06-05

**Authors:** Santiago Hors-Fraile, Francine Schneider, Luis Fernandez-Luque, Francisco Luna-Perejon, Anton Civit, Dimitris Spachos, Panagiotis Bamidis, Hein de Vries

**Affiliations:** 1 Department of Architecture and Computer Technology, Universidad de Sevilla, ETSII, Avenida Reina Mercedes S/N, 41012 Seville, Spain; 2 Department of Health Promotion, School for Public Health and Primary Care (Caphri), Maastricht University, P. Debyeplein 1, 6229 HA Maastricht, The Netherlands; 3 Qatar Computing Research Institute, Hamad bin Khalifa University, Education City, Doha, Qatar; 4 Salumedia Tecnologías, Avenida República Argentina 24, Edificio Torre de los Remedios, Planta 5, Módulo A, Seville, Spain; 5 Medical School, Faculty of Health Sciences, Aristotle University of Thessaloniki, Thessaloniki, Greece

**Keywords:** Recommender system, Tailored messages, Smoking cessation, Mobile app, Patient, mHealth

## Abstract

**Background:**

Smoking is one of the most avoidable health risk factors, and yet the quitting success rates are low. The usage of tailored health messages to support quitting has been proved to increase quitting success rates. Technology can provide convenient means to deliver tailored health messages. Health recommender systems are information-filtering algorithms that can choose the most relevant health-related items—for instance, motivational messages aimed at smoking cessation—for each user based on his or her profile. The goals of this study are to analyze the perceived quality of an mHealth recommender system aimed at smoking cessation, and to assess the level of engagement with the messages delivered to users via this medium.

**Methods:**

Patients participating in a smoking cessation program will be provided with a mobile app to receive tailored motivational health messages selected by a health recommender system, based on their profile retrieved from an electronic health record as the initial knowledge source. Patients’ feedback on the messages and their interactions with the app will be analyzed and evaluated following an observational prospective methodology to a) assess the perceived quality of the mobile-based health recommender system and the messages, using the precision and time-to-read metrics and an 18-item questionnaire delivered to all patients who complete the program, and b) measure patient engagement with the mobile-based health recommender system using aggregated data analytic metrics like session frequency and, to determine the individual-level engagement, the rate of read messages for each user. This paper details the implementation and evaluation protocol that will be followed.

**Discussion:**

This study will explore whether a health recommender system algorithm integrated with an electronic health record can predict which tailored motivational health messages patients would prefer and consider to be of a good quality, encouraging them to engage with the system. The outcomes of this study will help future researchers design better tailored motivational message-sending recommender systems for smoking cessation to increase patient engagement, reduce attrition, and, as a result, increase the rates of smoking cessation.

**Trial registration:**

The trial was registered at clinicaltrials.org under the ClinicalTrials.gov identifier NCT03206619 on July 2nd 2017. Retrospectively registered.

## Background

New technologies such as smartphones and wearables can be used to support behavior change among patients, as many studies have already shown [1–7]. One of the ways in which technology is used to do so is by designing tailored health messages targeted at patients. Some such platforms use expert systems [8] that use the rules of human expert reasoning and infer results based on people’s answers to questions about behavior knowledge and motivational aspects like attitude and self-efficacy. Yet another type of system is a recommender system, which aims at sending messages tailored to users’ preferences [9–11]. These platforms employ algorithms to predict which message is most similar to its users’ previously preferred messages.

**Fig. 1:**
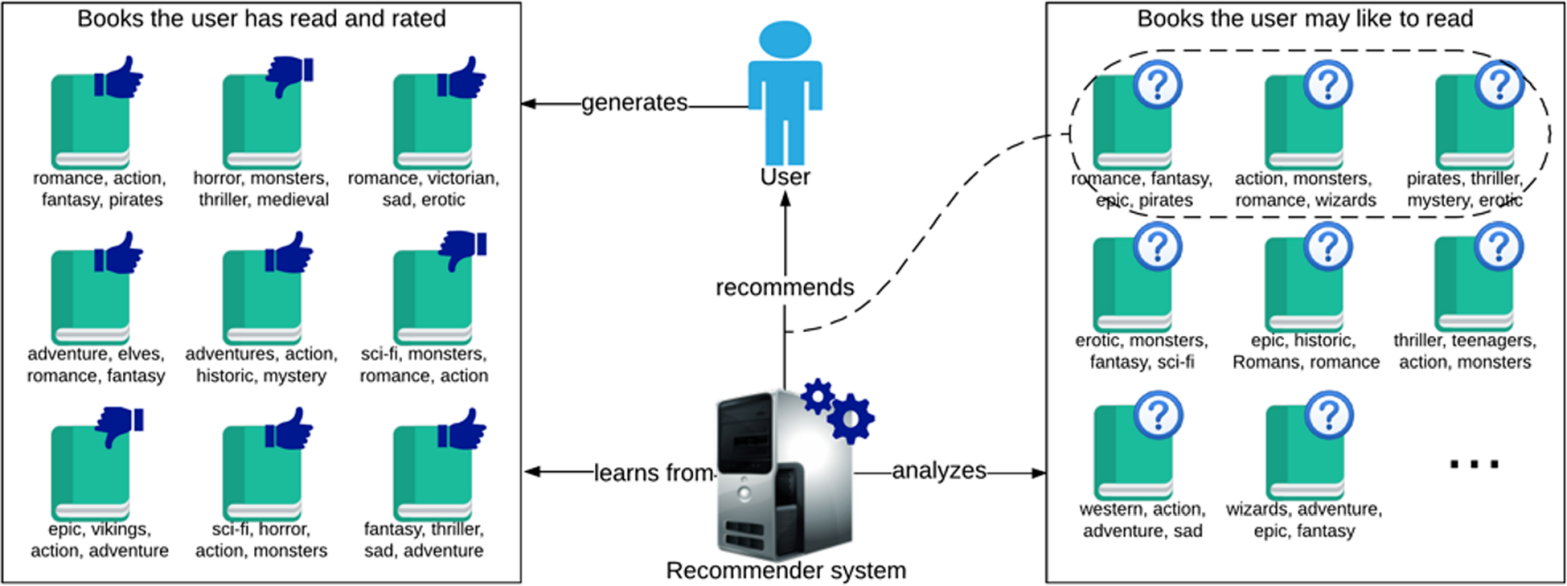
Concept diagram of a simple recommender system. In this example, the recommended items are books. The user receives suggestions on what to read next based on the genres and features of books he or she has liked in the

A recommender system is a piece of software that learns to predict the best item for each user from a set of items [12]. Items can be text messages, movies, books, people, or anything else that can be recommended. Recommender systems have been exploited most extensively in the spheres of e-commerce and leisure, through the recommendation of, for instance, movies, books, and music [13]. For example, if a system knows the books you have liked in the past, it aims to forecast the books you may also like in the future. This can be done using different techniques like comparing the features of the books you have liked with the features of other books you have not read, as shown in Fig. 1, or by considering books that people with similar tastes as you have also liked.

Recommender systems have also been used in the healthcare domain. Health recommender systems are especially aimed at providing feedback and recommendations on health status and heath behaviors, such as lifestyle, nutrition [14], obesity [15], diabetes [16, 17], drug side effects [18], and smoking cessation [19]. The type of feedback is based on algorithms predicting the type of message needed on the basis of previously measured variables. The required input can be taken from electronic health records as described by Wiesner et al. [20] or may be based on a person’s interest in specific leisure-time behaviors.

However, few studies use this technique, and their potential is still to be exploited [21–23]. In addition, health recommender systems need to be precise and accurate in order to be effective. That optimization can already be found in recommender systems used for ecommerce and leisure. However, assessing the quality of health recommender systems in terms of appreciation of the recommendations by patients and their behavioral effects has so far not been explored in-depth. This can be done using experimental designs aimed at measuring their objective accuracy [24] or by finding out their users’ subjective opinions as described in the ResQue framework [25]. The goal of this study is develop and evaluate the quality of a health recommender system to fill this existing gap.

The health recommender system chosen for the study is aimed at supporting smoking cessation, because smoking is directly associated with a number of diseases: chronic obstructive pulmonary disease, asthma exacerbation, cataracts, pulmonary fibrosis, oral cavity cancer, pharynx cancer, larynx cancer, esophagus cancer, lung cancer, and bladder cancer, among others [26]. Consequently, quitting smoking is the most important decision smokers can take to optimize their chances to reduce health risks and increase the longevity of their lives [27]. Several studies explored the effects of innovative tailored behavioral change methods for smoking cessation and demonstrated that they can contribute to a higher success rates [28–30]. Other studies showed that the delivery of tailored motivational messages using mobile phones as a platform could be an effective way to enable smoking cessation [31–35] as well as to impact other aspects of health, such as promoting physical activity and exercise [36, 37], mental healthcare [38], and alcohol-related harm [39], among others. These tailored messages are usually pieces of text based on the user’s responses to certain questions, assessing their attitude, social support, self-efficacy, and type of action planning.

Yet, quitting smoking is often accompanied by several barriers, such as nicotine abstinence syndrome that may have consequences like headaches, cravings, intestinal disorders, weight gain, insomnia, restlessness, nervousness, depression, and irritability, among others [40, 41]. As a consequence, the rate of smoking relapses is high and success rates are often low [42–46], illustrating a clear need for providing messages that are highly personalized and tailored to the needs of each person at that point in time. Health recommender systems may have specific added value here as they use algorithms to optimally choose not only the type of message to be sent but also when it is sent.

In the current study, we will combine the principles of tailoring with two recommender systems, which customize messages and predict the best time to send the tailored motivational health messages to a cohort of patients who are trying to quit smoking using a mobile app. To select the most relevant message topic that can foster healthier habits in each patient, a health recommendation system algorithm (HRSA) accesses each patient’s personal health records and feeds the algorithm with this health data. Since the messages are delivered using smartphones and are tailored as per each patient’s health data, we call our system an mHealth Recommender System (m-HRS).

Wendel et al. [47] suggested four basic requirements to reduce potential barriers to using recommender systems: 1. Effort input minimization to reduce users’ burden; 2. Privacy assurance; 3. Optimizing message usefulness; and 4. Enjoyment.

The quality of the system is strongly associated to the patients’ attitudes toward the system and, consequently, to its usage [48]. A health system that patients feel is not of a good quality and one they cannot trust will not be used [49]. By increasing the perceived quality of the system, patients will be encouraged to adopt the system and use it more, which will lead to better health outcomes [50, 51].

Attrition is a well-known problem in digital healthcare interventions [52]. It is important to increase appreciation and thus raise patient engagement, thereby keeping attrition to a minimum. This is especially relevant in m-HRS because the more user engagement with the system, the more the system learns about the user, and, consequently, the stronger the system becomes. Alkhaldi et al. explained the importance of user engagement in digital health interventions [53]. Although there are different definitions of engagement, the one used in this study was proposed by Alkhaldi et al. in the study “Users revisiting the digital intervention”. A high engagement in digital health interventions is also associated with better patient health outcomes [54, 55]. This is because engaged patients read more tailored motivational health messages and therefore receive more such prompts. This has a positive impact on their behavior change, such as quitting smoking, as some studies have already shown [56, 57]. However, the novelty effect may also influence their attrition. Consequently, it would be necessary to compare how patients’ opinions regarding the HRS messages evolve throughout the intervention.

In conclusion, the first goal of this study is to describe the patients’ perceived quality of the m-HRS. The second goal is to assess the patients’ level of engagement with the messages generated by the system. Identifying which system features and user characteristics determine differences in user engagement and quality perception may help improve the design of future m-HRS platforms.

## Methods/Design

### Participants

For this observational prospective study, we will analyze patients participating in the SmokeFreeBrain project [58] for a period of 12 months.

The inclusion criteria for the study are patients who are attending the smoking cessation program Smoke Free Brain [59, 60] at the Virgen del Rocio University Hospital, are at least 18 years old, are willing to start treatment to quit smoking, own an Android smartphone and know how to use it, have installed the smoking cessation app recommended by the doctor, and who have not previously had any known adverse effects to the pharmacological treatment.

### Trial design

Patients will be provided with a purpose-built Android-based mobile application that allows them to receive messages and rate them on their smartphones. The app is called “Libre de humos”, or “Smokefree” in English. From here onward, we will refer to it as “the app”. The app’s other features include a goal achievement dashboard, physical exercise records, a relapse diary, a relaxation tool, mini-games to help patients overcome cravings through distractions—one of which is based on the webFitForAll exergaming platform protocol [61, 62]—and an informative section with content on various topics related to smoking cessation.

As no formal methodology has yet been used to assess mobile health recommender systems, we will use the message ratings, patients’ app usage behavior, and an adapted set of questions for the patients to compare their levels of quality appreciation and engagement. This will allow us to compare differences between the m-HRS patients’ opinions over time.

### App features and clinical integration

The present study will focus on exploiting features of smartphones that allow users to receive messages from a server and track their activity and interaction. The app can connect with the smoking cessation unit of the hospital’s electronic health record using Mirth Connect software. It interconnects the following:

- The hospital user database to access the patients’ demographic data
- The hospital clinical data base to access the patients’ clinical information
- The Lightweight Directory Access Protocol to validate the credentials of the healthcare professionals who access the hospital system

The information requested by the app is processed and selected from the electronic health record database, formatted using the ISO13606 standard, and sent back by the Mirth Connect platform.

Therefore, all the information regarding a patient’s profile—name, age, gender, date of quitting smoking, type of pharmacological treatment—is automatically loaded in the app without patients needing to input anything apart from a code provided by their clinicians. Furthermore, using the clinical station—the system interface at the hospital—healthcare professionals can monitor the patients’ activity using the app. For instance, if a patient logs a relapse, his or her doctors will be able to access it, provided they have the patient’s consent to do so.

**Fig. 2:**
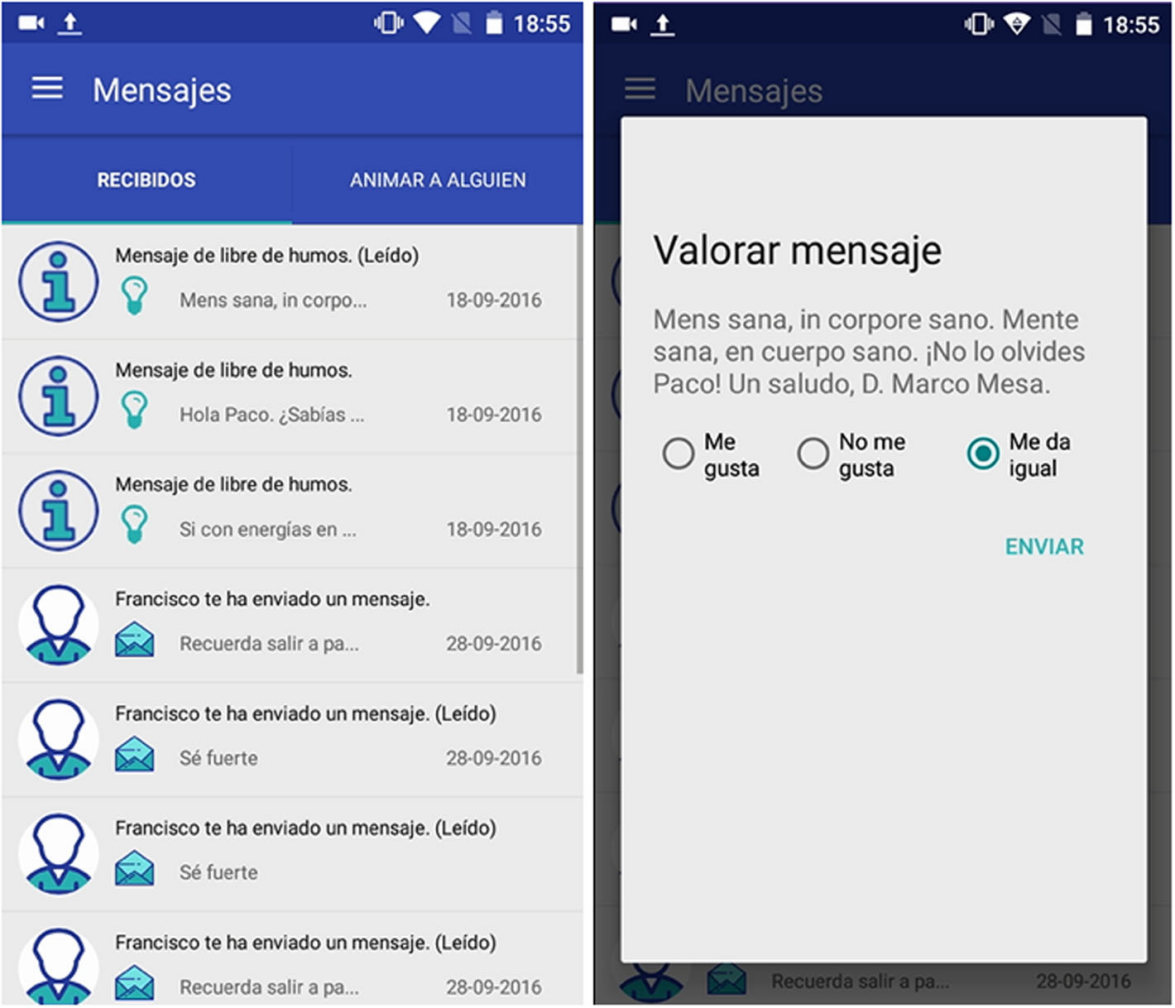
Screen captures of the message list and the rating mechanism of the “Libre de humos” smoking cessation app. Patients can provide feedback on the messages they receive via the app. They can provide feedback on each message by indicating “like”, “dislike”, or “indifferent”

The app will allow patients to rate the messages they have received using buttons to “Like” (positive), “Dislike” (negative), or “Don’t mind” (neutral), as shown in Fig. 2. The messages they will receive fall under one of the following five topics: general motivation, diet tips, physical exercise tips, personal performance, and the benefits of being a non-smoker.

Each of these topics has a pool of 150 different messages, along with useful tailored information for the patients. These topics and messages have been approved by a smoking cessation psychologist as well as a pulmonologist from the hospital.

Thus, this design will show whether patients prefer the messages selected by the m-HRS at the end of the pilot as a consequence of the HRSA being trained to better match the patient’s preferences, as opposed to the messages sent at the beginning when the HRSA was not configured to learn and improve messages based on the patients’ feedback.

#### User-centric considerations in the health recommender system

Based on Wendel et al.’s study [47] on fostering health recommendation systems, we took into account the following:

- Effort: Any effort required from the patient’s side has been minimized by removing the entry barrier of having to manually input their profile details. These details are automatically loaded from the hospital electronic health record.
- Privacy risk: The app only stores the patient’s name and all communication follows the MD5 encryption protocol as required to meet the hospital’s privacy requirements. The app and the electronic health record exchange XML documents following the HL7 protocol using the Mirth Connect engine.
- Usefulness: The topics of the messages and the messages themselves were validated by a psychologist and pulmonologist to make sure they contain relevant scientific information.
- Enjoyment: The use of friendly and familiar language combined with the frequency and timing of the messages makes the app feel personal and less robotic. This follows the gamification principle of “unpredictability and randomness” as per the Octalysis gamification framework [63].

Finally, Wendel et al.’s study also referred to the intermediary that introduces the system to the patients. In this case, it is the healthcare professional from the hospital’s smoking cessation unit who presents and endorses the m-HRS. This fact may also positively contribute to patients using it.

#### Choosing the message topic

**Fig. 3:**
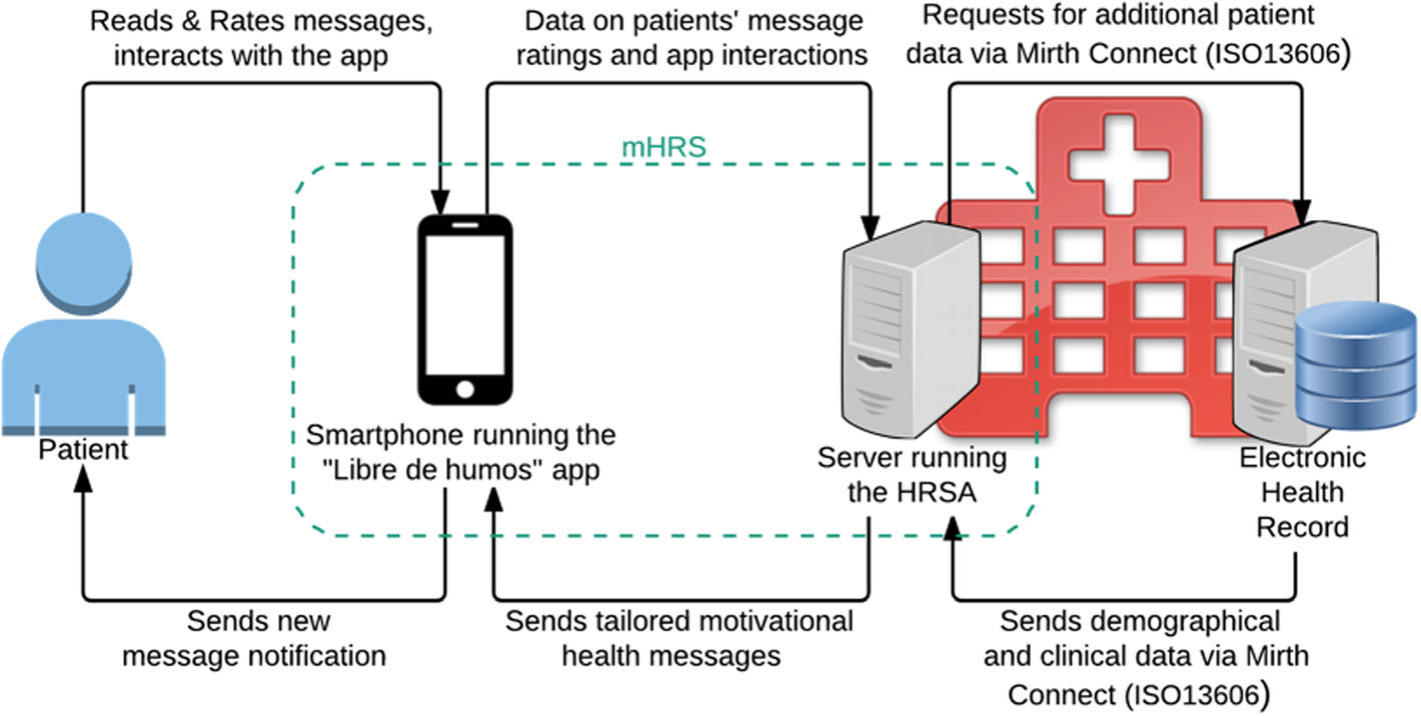
Description of the mHealth Recommender System’s architecture and flow of data information

The data on the message ratings and the patients’ interaction with the app will be sent to the hospital server, where it will be processed by an HRSA to enable it to choose the next message for each user as well as the time at which it should be sent. Figure 3 shows a conceptualization of this mHealth Recommender System architecture. The HRSA is an algorithm that follows a hybrid approach to improve its performance, as suggested by Burke [64]. The algorithm combines the following three factors to compute the results:

- Patient’s demographic similarity: Demographic influence will be based on the similarity of patients in terms of their age, gender, employment status, date they stopped smoking, and scores on the Fagerström [65] and Richmond tests [66].
- Perceived utility of the message topics: The utility of the messages will be measured as follows: Patients will be prompted to rate each message with “like”, “dislike”, or “neutral”. In case of multiple ratings given to the same message, the old ratings will be overridden. Visits to sections of the app on the same topic as the messages or re-read messages on a given topic will also be considered.
- Statement of initial interest: Patients need to fill out a questionnaire consisting of five questions in which they state their interest in the five message topics. This interest can be rated as positive, neutral, or negative. Patients can modify their answers anytime through the app.

Once the topic has been selected, the system selects a previously unsent message from the database and tailors it to the user so that it is more personalized. For instance, the message may include the name of the user.

#### Sending the messages

The frequency at which patients will receive messages is based on the conclusions of Abroms et al. [67] considered in combination with the transtheoretical behavior change model [68]. This model was chosen because the counseling offered at the hospital’s smoking cessation unit is partially based on it, and we wanted the messages to be in line with this counseling. We started considering patients in the preparation phase, which is when they can join the program.

- Preparation: One message a day until the day of quitting—the topic of these messages is not predicted by the HRSA but chosen at random, and they cannot be rated, so they have no influence on the HRSA
- Action: Four messages on the day of quitting, and one message a day for the following week.
- Maintenance: Three messages a week after that, for one year.

In the maintenance phase, as per the transtheoretical behavioral change model, the messages will be sent at random days during the week. In total, a patient who completes the yearlong treatment will receive a total of 150 messages from the m-HRS. The time at which the messages will be sent is also calculated by the HRSA, based on the time difference between when a message is sent and when it is read. The day will be divided in 12 two-hour periods during which the message can be sent at any random time. The sooner the patient reads the message after receipt, the more likely it is that the next message will be sent in the same two-hour period. Patients can deactivate messages during a specific period of time through a do-not-disturb setting. Just like the final decision of the message topic, the messages’ timing is also based on a probability vector.

### Outcomes

Our primary outcomes are the patients’ perceived quality of the m-HRS and their engagement with it. We define quality as a set of features that makes the system recommend timely and relevant messages for each patient. As no formal methodology has yet been used to assess the quality of and engagement with HRS, we will use both objective and subjective quality measures along with engagement metrics at an aggregated and individual level.

### Quality

#### Objective quality

Since the HRSA predicts the message topic and timing of delivery, the objective perceived quality will be measured using two metrics: the precision of the message topics, and how long after receiving the message the users read it.

The precision metric is defined as the relation between the number of hits per message in the first month (baseline) and the extent of “positive”, “neutral”, and “negative” feedback per message in month 3, month 6, month 9, and month 12. Hits are messages with only positive feedback (1) Precision_p_ and with both positive and neutral feedback (2) Precision_p&n_.

1 $$ Precisio{n}_P=\frac{\mid \mathrm{m} essages\ with\ positive\ feedback\mid }{\mid all\  messages\ with\  any\  feedback\mid } $$

2 $$ Precisio{n}_{p\&n}=\frac{\mid messages\ with\ positive\ and\ neutral\ feedback\mid }{\mid all\  messages\ with\  any\  feedback\mid } $$

We expect the acceptance rate to be higher in month 12, as compared to the previous months, as by this time the HRSA will have more patients and enough information to make accurate decisions.

We will calculate the precision of the system at different points of time, which will reflect the users’ feedback and their interactions with specific sections of the app. All this information is stored in the database of the m-HRS.

The time-to-read metric is defined as the time difference between when the message was sent and when it was read by the user. We will use it to assess the evolution in the quality of the HRSA to predict the best time at which to send a user messages—we theorize that the lesser the time-to-read, the better it is for the user to receive messages at that time.

### Subjective quality

The subjective quality will be evaluated through eighteen questions assessed using a five-point Likert scale answered by all patients at the end of their smoking cessation program. The questionnaire is adapted to the context of smoking cessation and to fit within the expected time frame in which the patients will have to complete it during their consultation at the hospital. The first twelve questions are a selection of those proposed in the ResQue framework [25] intended to measure the quality of the user’s experience with a recommender system and its influence on the user’s behaviors and intentions. The remaining six questions are designed on the basis of the i-Change behavior change model [69–71] to identify whether the messages also have an impact on the patients’ motivations.

### Engagement

#### Engagement at an aggregated level

This will be measured through five factors using the analytics software Yahoo Flurry, which is integrated in the m-HRS app. As described by the software developers themselves [72], these factors are as follows:

1. Rolling retention: The percentage of users still active N days after installation. This is a ratio of the number of users whose last day of activity is past day N to the number of users who could have been active on day N (i.e., the sum of new users up until day N).
2. Session length distribution: The session length is defined simply as the length of time between the start app event and the end app event. The session length determines engagement in terms of how much time patients spend on the app per session.
3. Session frequency: Frequency of Use is a measure of how often each unique user used the app within a given time interval.
4. Sessions per user: A session is defined as one use of the app by a patient. This begins when the application is launched and ends when the application is terminated.
5. Return rate: Return rate measures the percentage of users who return to the app at a specific time after installation. We look at this value by cohort group, that is, based on when users first opened the app. It is calculated as the ratio of the number of users active on a given day, week, or month to the size of the cohort. The install date is considered as Day 0. For example, the Return Rate for day 7 is the percentage of users that opened the app on the 7th day after installation. User activity on day 8, 9, and so on does not impact this value.

#### Engagement at an individual level

This will be assessed based on the rate of messages read by the user. It is calculated as the ratio of the messages a user has read and the total number of messages the system has sent to the user.

### Sample size

We aim to recruit 120 patients. No power analysis was done for this study, as we followed a convenient sample [73] method: our recruiting partner Hospital Virgen del Rocío estimated they could only access 120 patients within the time limits of the project. The expected dropout rate is 50%. This means the system will have sent at least 9000 messages to patients before the study ends (a minimum of 60 patients ending the treatment multiplied by 150 messages sent throughout the treatment).

### Ethics

The Social Local and Mobile intervention this study is based on has been approved by the Ethical Committee of the Virgen del Rocío and the Virgen Macarena University Hospitals (approval number SFB-APP_EC-2016-01). The research activities will strictly follow hospital regulations. Regarding the use of data, this project will conform to the regulations of the Personal Data Act (based on the European Data Protection Directive).

## Discussion

This study will explore how patients enrolled in a smoking cessation program perceive and engage with an m-HRS that sends them periodic motivational messages encouraging them to stop smoking. We also expect the m-HRS to help increase the low attrition rate that is typically seen in eHealth trials [74].

The answers to this study’s research questions will benefit future recommender system-based behavioral change interventions. Researchers will be able to re-use our proposed perceived quality and engagement metrics and exploit the knowledge derived in order to design new and improved health recommender systems that take into account user appreciation and engagement. Thus, the health messages sent to the users will be more interesting, relevant, and engaging for them.

We expect the patients’ perceived quality of the system to increase over time, that is, for the precision of the messages to increase and the patients’ time to read the messages to decrease. However, we should consider that, at some point during the intervention, patients may stop reading the messages because they have uninstalled the app. This may be because they have successfully quit smoking and do not want any smoking-related reminders, because they have relapsed, or because they never engaged with the app in the first place and have decided to remove it from their smartphone. Therefore, these metrics will need to be carefully analyzed to avoid simplistic conclusions.

The users’ in-app activity analytics will provide an insight into how patients react to the messages the HRSA selects for them. This opens up several future research opportunities, such as comparing whether the theoretical interests patients claim to have are in fact their interests, noting the time they stop rating messages because they are no longer interested in receiving more messages, and having an initial m-HRS-generated message patient acceptance data report for future implementation in smoking cessation programs.

However, this study may suffer from the trust problem like all recommender agents. Despite the elaborate design of the HRSA, should a patient receive a single message that is out of context, it may ruin her confidence in the system and she may stop using the m-HRS altogether [75]. In order to minimize this, the messages have been designed such that they are applicable to almost all circumstances and experiences of a patient.

The premise behind this study using an m-HRS instead of a standard initial questionnaire of an expert system is the high on-the-go adaptability potential for patients without bothering them with questionnaires to update their preferences. Another reason for this is the potential scalability and robustness the m-HRS can achieve in the long term when the HRSA database contains information on a large number of patients. This will provide the basis for health behavior change studies to be conducted at a larger scale and to become more advanced since the recommendations for patients will be selected from a more complex set of options.

### Limitations and generalizability

This study is limited by the design of the HRSA, which cannot distinguish between longitudinal interest changes in the patient cohort during the smoking cessation process. In addition, the HRSA is also limited because it does not differentiate between working days and holidays.

The imposed maximum sample size and the lack of a-priori power analysis may also be a limiting factor for the generalizability of the results of the study. However, to minimize this, we will do power calculations for minimum detectable effect sizes for key outcomes.

The patient engagement analysis is limited because it is not possible to cross-compare the individual-level engagement metrics to the aggregated-level engagement metrics.

Since the study will only be conducted in the city of Seville, it may not be possible for the outcomes to be directly generalized to the Spanish population as a whole.

## Abbreviations

HRSA: Health recommendation system algorithm
m-HRS: mHealth Recommender System
